# Isolation and Characterization of E8 Monoclonal Antibodies from Donors Vaccinated with Recombinant Vaccinia Vaccine with Efficient Neutralization of Authentic Monkeypox Virus

**DOI:** 10.3390/vaccines13050471

**Published:** 2025-04-27

**Authors:** Yutao Shi, Shuhui Wang, Yanling Hao, Xiuli Shen, Jun Zhang, Shuo Wang, Junjie Zhang, Yuyu Fu, Ran Chen, Dong Wang, Yiming Shao, Dan Li, Ying Liu

**Affiliations:** 1 National Key Laboratory of Intelligent Tracking and Forecasting for Infectious Diseases, National Center for AIDS/STD Control and Prevention, Chinese Center for Disease Control and Prevention, Beijing 102206, China; 17356674396@163.com (Y.S.); shuhuiwang@chinaaids.cn (S.W.); haoyl@chinaaids.cn (Y.H.); zhangjunn_ah@163.com (J.Z.); wswangshuo1029@126.com (S.W.); zhangjj0673@163.com (J.Z.); fuyuyu11877@163.com (Y.F.); chenran868686@163.com (R.C.); wangdong19971229@163.com (D.W.); 2 Changping Laboratory, Yard 28, Science Park Road, Changping District, Beijing 102206, China; shenxiuli459@163.com (X.S.); yshao@bjmu.edu.cn (Y.S.)

**Keywords:** monkeypox virus, vaccinia virus, neutralizing antibody, E8 protein

## Abstract

Background/Objectives: Monkeypox, twice declared a public health emergency of international concern by the WHO, currently lacks approved targeted therapeutics. This study focused on the development of monkeypox virus (MPXV) E8-specific human monoclonal antibodies (mAbs) derived from recipients of the recombinant vaccinia vaccine (rTV), with subsequent evaluation of their cross-neutralizing activity against orthopoxviruses, including the vaccinia virus (VACV) and MPXV. Methods: Three mAbs (C5, C9, and F8) were isolated from rTV vaccinees. Structural mapping characterized their binding domains on the MPXV E8 and VACV D8 proteins. Neutralization potency was assessed against the VACV TianTan strain and MPXV clade IIb. A combo was further evaluated in a VACV-infected mice model for clinical recovery and viral load reduction. Complement-dependent enhancement mechanisms were also investigated in vitro. Results: C9 targets the virion surface region of E8 and both the virion surface region and intravirion region of D8, showing cross-neutralization activity against the MPXV (IC_50_ = 3.0 μg/mL) and VACV (IC_50_ = 51.1 ng/mL) in vitro. All three antibodies demonstrated potent neutralization against the VACV in vitro: C5 (IC_50_ = 3.9 ng/mL), C9 (IC_50_ = 51.1 ng/mL), and F8 (IC_50_ = 101.1 ng/mL). Notably, complement enhanced neutralization against the VACV by >50-fold, although no enhancement was observed for the MPXV. In vivo administration accelerated clinical recovery by 24 h and achieved significant viral clearance (0.9-log reduction). Conclusions: E8-targeting mAbs exhibited broad-spectrum neutralization against orthopoxviruses, demonstrating therapeutic potential against both historical (VACV) and emerging (MPXV) pathogens. However, MPXV’s resistance to complement-dependent enhancement highlights the necessity for pathogen-adapted optimization. These findings establish E8 as a critical conserved target for pan-poxvirus VACV and MPXV countermeasure development.

## 1. Introduction

Monkeypox (mpox) is a zoonotic disease caused by the monkeypox virus (MPXV), which belongs to the orthopoxvirus genus within the Poxviridae family [1]. It is categorized into two distinct clades: clade I, which is primarily distributed in Central Africa, and clade II, mainly found in West Africa [2,3]. The IIb clade was responsible for the global Mpox outbreak in 2022. On 23 July 2022, the World Health Organization (WHO) declared mpox a public health emergency of international concern (PHEIC) [4,5]. As of February 2025, 131 countries and regions have reported over 130,000 confirmed cases of mpox to the WHO, with fatalities reaching 304 [6]. On 14 August 2024, due to a surge in clade I infection cases in Central Africa, the WHO once again declared mpox a PHEIC [7,8].

Mpox is usually self-limiting in individuals with normal immune function, and the typical clinical symptoms include lymphadenopathy, fever, and rash [9]. However, in immunocompromised populations and children, infection with the MPXV may lead to severe consequences [10,11,12,13].

Treatment for mpox is primarily supportive, as no specific antiviral medications have been approved to date. Tecovirimat, originally developed as a drug for smallpox bioterrorism threats, has been considered a promising therapeutic option for mpox [14]. However, the results of a phase II trial (ClinicalTrials.gov ID: NCT05559099) indicated that tecovirimat did not reduce the duration of lesions in children and adults with clade I MPXV infection [15]. Meanwhile, other studies have reported that the long-term use of tecovirimat in HIV-positive individuals with severe immunosuppression and co-infection with the MPXV may lead to drug resistance [15,16]. Therefore, the development of treatments for mpox is imperative. Monoclonal antibodies (mAbs) have been widely utilized in the treatment and prevention of infectious diseases [17]. The development of mAbs specific to MPXV surface membrane proteins is crucial for drug development [18].

The MPXV is classified in the same genus as the variola virus (VARV) and vaccinia virus (VACV), sharing a high level of homologous sequences [19]. Due to the cross-reactivity between the VACV and MPXV, a VACV-based smallpox vaccine conferred approximately 85% protection against the MPXV [20,21]. Early studies have demonstrated that neutralizing antibodies induced by VACV surface membrane proteins (such as A27, L1, D8, etc.) exhibit strong antiviral activity against orthopoxviruses [22,23,24,25,26]. Several antibodies targeting the surface membrane proteins of the MPXV have demonstrated significant protective effects in both in vitro and animal models [27,28,29,30,31,32].

Similar to the VACV, the MPXV also has two forms of infectious virions, an intracellular mature virion (IMV) and an extracellular enveloped virion (EEV) that is generated when the IMV is encapsulated by endosomal membranes. The IMV, remaining in the cell until cell lysis, is highly infectious and stable, making it suitable for spreading infections between hosts [33]. Among IMV surface proteins capable of eliciting neutralizing antibodies, E8 has emerged as a critical target due to its dual role in viral entry and immune evasion, as well as the fact that it shares high homology with VACV D8, the protein essential for VACV cellular entry [24].

In this study, we observed that the sera of the recombinant vaccinia vaccine (rTV) exhibited significantly higher E8-binding ability compared to D8. Based on this finding, we used the labeled MPXV surface membrane protein E8 as the probe to screen MPXV-specific memory B cells from their PBMCs, identifying three fully human mAbs that target E8. These antibodies exhibited strong neutralizing and protective effects both in vitro and in vivo, as well as combined.

## 2. Materials and Methods

### 2.1. Viruses, Cells, and Mice

The VACV Tiantan strain (VTT) and a recombinant Tiantan strain expressing GFP (TT-GFP) were preserved by the National Center for AIDS/STD Control and Prevention, China CDC (NCAIDS/China CDC) and propagated and titered in primary chicken embryo fibroblasts (CEFs). The MPXV (Clade IIb) was provided by the Beijing Center for Disease Prevention and Control and the National Institute for Communicable Disease Control and Prevention, China CDC. The VACV and MPXV were manipulated under BSL-2 and BSL-3 conditions, respectively. Human embryonic kidney (HEK) Expi293F cells were cultured at 37 °C in an SMM 293-TII expression medium supplemented with 5% CO_2_ in a shaking incubator at 200 rpm. HEK 293T and Vero cells were maintained at 37 °C in DMEM supplemented with 10% fetal bovine serum (FBS) and 1% penicillin–streptomycin (10,000 U/mL) (Gibco^TM^). All animal experiments were meticulously carried out in adherence to the pertinent animal welfare guidelines and regulations, receiving authorization through the China CDC Application for Ethical Approval for Research Involving Animals (approval number: 2024-CCDC-IACUC-009). Six-week-old female BALB/c mice were purchased from Beijing Vital River Laboratory Animal Technology Co., Ltd. (Beijing, China). The experiments were conducted at the Laboratory Animal Center of China CDC within an animal biosafety level 2 facility.

### 2.2. Donors

HIV-negative healthy adult donors enrolled in the clinical trial (registered at the Chinese Clinical Trial Registry, ChiCTR1900021422), approved by the NCAIDS and Beijing Youan Hospital IRBs, received two doses of the HIV candidate vaccine rTV, a recombinant VTT expressing HIV antigens. The HIV antigens were inserted into the thymidine kinase (TK) gene locus of the VTT, allowing for the normal expression of other vaccinia genes while delivering the HIV antigens. We assayed the E8-specific binding antibody and neutralizing antibody against the VTT of 140 serum samples collected from donors. Since serum from donor 1R073 exhibited the highest E8-binding and VACV-neutralization ability, his PBMCs were chosen for further single B cell sorting (Figure S1).

### 2.3. Antigen-Specific Single B Cell Sorting

Before sorting, thawed PBMCs were stained with a mixture of antibodies, including anti-CD3-Pacific Blue, anti-CD8-Pacific Blue, anti-CD14-Pacific Blue, anti-CD19-BV510, anti-CD20-ECD, anti-CD27-APCCy7, anti-IgG-FITC, anti-IgM-PercpCy5.5, anti-PD-1-PECy7, anti-CXCR5-APC-R700, CXCR3-PECy5, anti-CD45RA-BV650, and anti-CD4-BV605. The sorting probe was an MPXV E8 protein with His and Avi tags (purchased from ACROBiosystems, Beijing, China) and labeled with streptavidin-allophycocyanin (SA-APC) and streptavidin-R-phycoerythrin conjugates (SA-PE) (Invitrogen, Carlsbad, CA, USA). Cell viability was assessed using the LIVE/DEAD Fixable Dead Cell Stain Kit (Pacific Blue) (Invitrogen) to exclude dead cells. Utilizing a previously established platform, CD3-CD8-CD14-CD19+CD20+CD27+IgG+IgM-E8 + single B cells were sorted from the samples using flow cytometry (FACS Aria SORP, BD Biosciences, San Jose, CA, USA). The sorted single B cells were stored in a 96-well PCR plate containing lysis buffer and kept at −80 °C. Data analysis was conducted using FlowJo software version 10 (TreeStar, Ashland, OR, USA, 35354).

### 2.4. Single B Cell RT-PCR, Family Analysis, and Construction of the Vector

RT-PCR was employed to amplify the heavy and light chains of immunoglobulins, which were subsequently cloned into expression vectors to generate complete IgG 1 [34,35]. The 96-well PCR plate containing frozen, lysed single B cells was thawed at room temperature. Through RT-PCR, the heavy and light chains of the antibodies were amplified, and the resulting PCR products were sequenced. The sequences were analyzed using the IMGT database (www.imgt.org/IMGT_vquest/vquest) (accessed on 11 September 2024) to characterize the variable regions of the heavy and light chains. The variable region genes were then separately cloned into full-length IgG1 heavy chain and light chain expression vectors containing constant region sequences [34].

### 2.5. Co-Transfection of Heavy and Light Chains of Antibodies in 293T Cells and ELISA Screening for Specific Antibodies

The heavy- and light-chain plasmids of the constructed antibodies were co-transfected into HEK-293T cells using Lipofectamine 2000 (Invitrogen). Cells were cultured at 37 °C with 5% CO_2_ for 48 h. Subsequently, the cultured plates were subjected to 3 freeze–thaw cycles at −80 °C to 37 °C, and then centrifuged at 2000 rpm for 10 min. Following this, 10 µL of the supernatant was transferred to high-binding ELISA plates (Corning, Glendale, AZ, USA) coated with the E8 protein, and antibodies exhibiting positive ELISA results were selected for expression and purification.

### 2.6. Expression and Purification of Monoclonal Antibodies

Using PEI 25K (Polysciences, Warrington, PA, USA), equal amounts of heavy- and light-chain plasmids were co-transfected into 293F cells and cultured at 37 °C with 8% CO_2_ for 5 days. Subsequently, the cell culture supernatant was centrifuged at 125,000 rpm for 2 h, and the supernatant was filtered using a vacuum centrifuge. The filtered supernatant was then passed through a protein A column, and antibodies were eluted using a Gly-HCl elution buffer (Sigma-Aldrich, St. Louis, MO, USA). The antibodies were concentrated using 30 kDa concentrators and filtered through a 0.45 µm bacterial filter. Finally, the antibody concentration was measured using a spectrophotometer (Nanodrop 2000) (Thermo Fisher Scientific, Waltham, MA, USA).

### 2.7. Enzyme-Linked Immunosorbent Assay (ELISA)

High-binding ELISA plates were coated with 1 µg/mL of the MPXV E8 protein (Sangon Biotech, Shanghai, China) and the VACV D8 protein (AntibodySystem, Paris, France) overnight at 4 °C, respectively. The plates were incubated with mAbs, which were serially diluted 4-fold from an initial concentration of 5 µg/mL at 37 °C for 2 h. Following 5 washes, an HRP-conjugated goat anti-human IgG antibody (SouthernBiotech, Birmingham, AL, USA) diluted at 1:2000 was added and incubated for 1 h at 37 °C. After 5 final washes, the TMB substrate was applied and incubated at room temperature in the dark for 15 min, after which the reaction was terminated with 2 M sulfuric acid. The absorbance was measured at 450 nm with a reference wavelength of 630 nm using a microplate reader. Duplicate wells were included for each sample to ensure the accuracy of the results.

### 2.8. Biolayer Interferometry (BLI)

Antibody–antigen kinetics were measured using the Octet^®^ Red 96 system (ForteBio, Fremont, CA, USA). MPXV E8 and VACV D8 proteins were first diluted to 5 μg/mL in PBST (containing 0.02% Tween 20 and 0.1% BSA) and immobilized on AR2G biosensors (ForteBio, Germany) for 600 s. After a washing step with PBST for 120 s, the sensor tips were immersed in wells containing serially diluted antibodies (500 nM, 250 nM, 125 nM, 62.5 nM, 31.25 nM, 15.63 nM, and 7.82 nM) for 200 s to allow binding, followed by a 300 s dissociation step. A tandem method is employed for a competition assay. The antigen was saturated with the primary antibody for 300 s. Subsequently, the secondary antibody was introduced as the competing antibody, and the competition phase continued for another 300 s. The equilibrium dissociation constant (KD) values were calculated using a 1:1 binding model in Data Analysis Software 9.0.

### 2.9. Molecular Docking

The MPXV E8 protein, VACV D8 protein, and the antibody fragment variable (Fv) regions of C5, C9, and F8 were predicted by AlphaFold 3 [36]. Docking simulations were performed using Discovery Studio 2019 (BIOVIA, San Diego, CA, USA). The E8 protein structure was pre-processed in Discovery Studio by removing solvent molecules and optimizing the structure. The Fv regions were modeled using the Prepare Protein tool, followed by energy minimization. Docking was performed with the ZDOCK tool, using FFT-based docking to generate 1000 conformations for each antibody–antigen complex, using RDOCK to further improve binding stability for the top 10 docked models. The best model for each complex was selected based on the docking scores, and figures were prepared in PyMOL [37].

### 2.10. VACV IMV and EEV Neutralization Assays

For the VACV IMV neutralization assay, 25 µL of TT-GFP IMV (4 × 10^4^ PFU/mL) was incubated with mAbs, serially diluted 5-fold from an initial concentration of 10 µg/mL, at 37 °C for 2 h. The mAb–IMV mixture was then added to Vero cells and incubated with 5% CO_2_ at 37 °C for 2 h. After this, the mixture was removed and 100 µL of culture medium containing 0.5% methylcellulose was added to each well. The plates were incubated at 37 °C for 24 h.

To conduct the EEV neutralization assay, EEVs were harvested freshly. Vero cells cultured in T75 flasks with 90% confluence were infected with TT-GFP at 2 MOI for the preparation of the VACV EEV. After 2 days, the culture supernatant containing the EEV was harvested and then centrifuged twice at 450× *g* for 8 min to remove cell debris. The harvested EEV was incubated with 5-fold serial dilutions of mAbs at 10 µg/mL at 37 °C for 2 h and then added to Vero cells for infection as described above. The number of plaques was counted using the BIOREADER 4000 (BIOSYS, Miami, FL, USA). The half-maximal inhibitory concentration (IC_50_) values were determined using GraphPad Prism 10.1.2.

In the complement-dependent neutralization assay, an additional step involved first mixing the mAbs with 0.1% guinea pig complement in equal proportions. Negative controls comprised the VACV incubated with 0.1% guinea pig complement without the E8 antibody.

### 2.11. MPXV Neutralization Assay

The MPXV neutralization assay was performed on Vero cells using the plaque reduction assay. All experimental procedures were conducted within a BSL-3 laboratory. Firstly, 180 µL of the MPXV (1000 PFU/mL) was mixed in equal proportions with antibodies that had been serially diluted 10-fold from an initial concentration of 100 µg/mL. The mixture was incubated at 37 °C for 2 h. Following incubation, the antibody–virus mixture was transferred to Vero cells and further incubated at 37 °C with 5% CO_2_ for 1 h. Afterward, 100 µL of culture medium containing 0.5% methylcellulose was added, and the cells were cultured at 37 °C for 3 days. The results were determined by staining with a 0.2% crystal violet solution. A complement-dependent neutralization assay was also performed.

### 2.12. Protection Studies in Mice

Twelve 6–8-week-old BALB/c mice per group were deeply anesthetized with 1.25% tribromoethanol and subsequently infected via intranasal administration with 1 × 10^5^ PFU of the VTT. Then, the mice were intraperitoneally injected with either E8 mAbs at a dose of 10 mg/kg or PBS at 4 and 72 h post-infection. The combo (C5+C9+F8) was administered at a total dosage of 10 mg/kg, with each antibody constituting one-third of the total. Four days after infection, the lungs of half of the mice were collected for viral load assays. The other mice were monitored daily for mortality and changes in body weight for 14 consecutive days. Animals that lost more than 30% of their initial body weight were euthanized following the animal ethics protocols.

### 2.13. Viral Load

Lung tissue was weighed and then homogenized in 1 mL of 2% DMEM medium. The homogenate was centrifuged to collect the supernatant. DNA was extracted using the QIAamp DNA Blood Mini Kit (QIAGEN, Venlo, The Netherlands) and stored at −80 °C until use. The viral load was quantified using the ABI 7500/7500 Fast Real-time PCR System. Plasmid p-A27 carrying the VTT A27 gene was serially diluted (from 1 × 10^7^ to 10^1^ copies/µL) and sample DNA was used as the template for amplification. Forward and reverse primers, along with a TaqMan probe, were added at a final concentration of 10 µM in each reaction. The amplification protocol was as follows: 50 °C for 2 min, 95 °C for 30 s, 95 °C for 5 s, and 55 °C for 30 s, with 45 cycles. The TaqMan probe (5′-FAM-CTTCTTCAGATCCACACGAGAAA-MGB-3′) targeted the VTT A27L gene. The forward primer sequence was 5′-AGAACTAGAACGTGATCGG-3′, and the reverse sequence was 5′-TTTATCCTCAGTCATCAACGG-3′.

### 2.14. Statistical Analyses

GraphPad Prism software version 10.1.2 (GraphPad Software, Inc., San Diego, CA, USA) was used to perform statistical analyses. Non-parametric Mann–Whitney U tests with Bonferroni correction were applied for inter-group comparisons of body weight loss. The Kruskal–Wallis test was used to compare the viral load across groups. A *p*-value of <0.05 indicated a statistically significant difference.

## 3. Results

### 3.1. Isolation of E8-Specific IgG+CD19+ Cells

**Figure 1:**
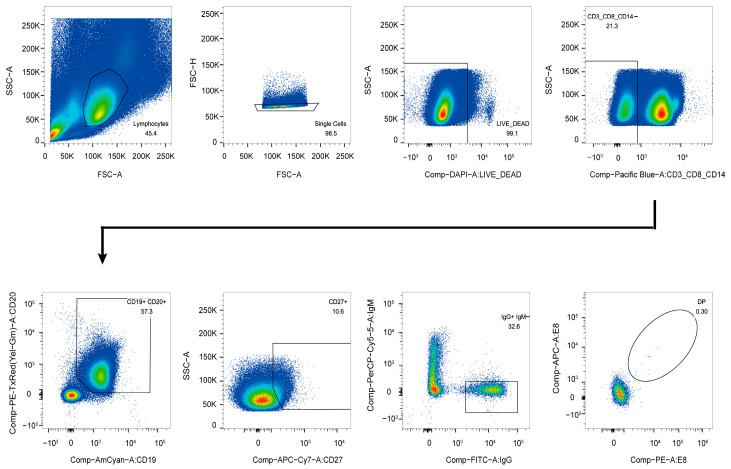
Isolation of E8-specific memory B cells from the PBMCs of vaccinated individuals using flow cytometry. Memory B cells (CD19+, CD20+, and IgG+) that bound to the E8-APC/PE probe were sorted into 96-well plates containing the lysate. The percentage of IgG+ B cells reactive to E8 is indicated.

Due to the high homology between the surface membrane proteins of the MPXV and VACV, we successfully isolated 54 E8-specific IgG+CD19+ B cells from the PBMCs of a donor receiving the rTV vaccine using the MPXV E8 protein as a molecular probe (Figure 1). From these 54 single B cells, we amplified 31 pairs of matched variable region genes for both the heavy and light chains. The detailed information regarding these genes is presented in Figure S2. These genes were subsequently cloned into full-length IgG1 expression vectors for further analysis.

### 3.2. The mAbs Specific for the MPXV E8 Protein

**Figure 2:**
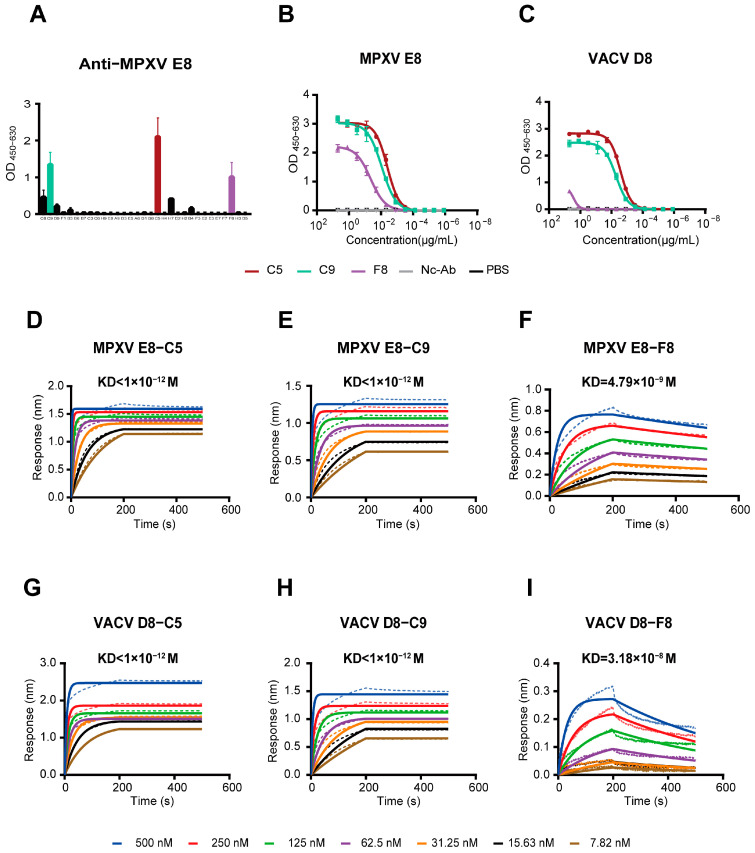
Binding activities and kinetics of the mAbs C5, C9, and F8 against MPXV E8 and VACV D8 proteins. (**A**) Screening results showing the binding activities of C5, C9, and F8 to MPXV E8. (**B**,**C**) ELISA analysis of MPXV E8 mAbs binding to MPXV E8 (**B**), and VACV D8 proteins (**C**). (**D**–**F**) Binding kinetics of MPXV mAbs to the MPXV E8 protein. (**G**–**I**) Binding kinetics of MPXV E8 mAbs to the VACV D8 protein.

To obtain functional mAbs, we conducted an ELISA to assay the E8-binding ability of mAbs by co-transfecting Vero cells with the matched heavy and light chains of the antibodies. From this initial screening, we successfully identified three mAbs—C5, C9, and F8—with notable binding activity (OD_450–630_ > 1.0) to the MPXV E8 protein (Figure 2A).

These three mAbs were subsequently expressed in 293 F cells and purified. A reducing SDS-PAGE analysis (Figure S2) confirmed the presence of the heavy-chain proteins at approximately 55 kDa and light-chain proteins at around 25 kDa, indicating the successful expression and proper assembly of the antibodies.

### 3.3. Analysis of the Variable-Region Sequences of Monoclonal Antibodies

To elucidate the molecular basis determining the specificity and affinity of the isolated mAbs, we performed a comprehensive analysis of the variable (V)-region sequences of both heavy and light chains. The detailed information is summarized in Table 1.

The heavy chains of the mAbs exhibit diverse V(D)J recombination events, as evidenced by their variable (IGHV), diversity (IGHD), and joining (IGHJ) gene segment usage. Notably, all heavy chains are derived from distinct IGHV genes: IGHV1-2*02, IGHV4-34*01, and IGHV1-24*01. This diversity suggests a broad repertoire, potentially enabling the recognition of varied epitopes. The complementarity-determining region 3 (CDR3) sequences of C5H were “ARTPPDLSGFH” and “ATAVYCGGDCYSPWFDP” in F8H, indicating diverse structural conformations. The somatic hypermutation (SHM) rates in the heavy chains range from approximately 4.86% to 5.56%, reflecting ongoing affinity maturation processes. These SHM levels are indicative of a robust adaptive immune response, potentially enhancing the binding affinity and specificity of the antibodies toward their viral targets [38,39,40,41].

The light chains further contribute to the antigen-binding specificity through their distinct V and J gene segment usage. The identified IGKV genes—IGKV2-28*01, IGKV1-39*01, and IGKV3-15*01—are associated with diverse binding properties. The V gene of C5L belongs to IGKV2-28*01, a member of the Vκ2 family. It has nine amino acids in CDR3. These characteristics may confer enhanced suitability for targeting small molecular epitopes or linear antigens. In contrast, IGKV1-39*01 (Vκ1 family) of C9L, with nine amino acids in CDR3, exhibits high combinatorial diversity, making it structurally optimized for recognizing conformational epitopes on antigens. Conversely, F8L utilizes IGKV3-15*01 (Vκ3 family) and has 10 amino acids in CDR3. This configuration is hypothesized to potentiate electrostatic complementarity toward negatively charged antigenic surfaces. These genes may complement the heavy chains’ variability to fine-tune antigen recognition.

The SHM rates in the light chains vary more significantly, with C5L exhibiting a higher mutation rate of 5.92% compared to C9L and F8L, which were both at 2.94%. This disparity may reflect differential selective pressures during B cell maturation, possibly influenced by the specific epitopes targeted by each antibody.

### 3.4. Three MPXV Monoclonal Antibodies Exhibit Strong Binding Activity

To evaluate the binding efficacy of the mAbs against the MPXV E8 and VACV D8 proteins, we employed ELISA and BLI assays. As detailed below, the three mAbs—C5, C9, and F8—showed distinct binding profiles. The ELISA results revealed that C5 and C9 exhibited strong binding affinities towards MPXV E8 (EC_50_ = 4.045 ng/mL and 8.903 ng/mL, respectively), whereas F8 displayed weaker binding (EC_50_ = 45.11 ng/mL) (Figure 2B). In contrast, when evaluating binding to VACV D8, C5 (EC_50_ = 2.632 ng/mL) and C9 (EC_50_ = 5.153 ng/mL) maintained their strong binding ability, while F8 displayed a >500-fold reduction in affinity (EC_50_ = 2.672 ng/mL) (Figure 2C).

BLI quantification confirmed the ELISA-derived binding hierarchy, providing a kinetic resolution of mAb–antigen interactions. As shown in Figure 2D–I, C5 and C9 exhibited near-picomolar affinity for both MPXV E8 and VACV D8, with KD values below 10^−12^ M (Table S1). Such ultrahigh affinity is indicative of a near-irreversible binding interaction, which is highly desirable for therapeutic applications where sustained antigen targets are critical. In contrast, F8 exhibited binding that was three orders of magnitude weaker (KD = 4.79 × 10^−9^ M), suggesting limited binding ability against the target proteins under physiological conditions. The high affinity of C5 and C9 may be attributed to their specific variable region characteristics, including CDR structure and favorable SHM profiles, as previously analyzed.

### 3.5. Potent Neutralizing Activity of the C9 mAb and Its Combination Against the Authentic MPXV

The neutralization results of the MPXV demonstrated that C5 and F8 exhibited no neutralizing activity, regardless of the presence of complement, suggesting that these antibodies target the non-neutralizing epitopes of MPXV E8. In contrast, C9 showed moderate neutralization activity, with IC_50_ values of 3.0 μg/mL (with complement) and 3.2 μg/mL (without complement). Interestingly, the combination of C5+C9+F8 showed no significant complement-independent synergistic effects, with IC_50_ values of 2.8 μg/mL in the presence of complement and 0.4 μg/mL in the absence of complement (Table 2).

### 3.6. All Three MPXV Monoclonal Antibodies Were Able to Neutralize the VACV IMV In Vitro

**Figure 3:**
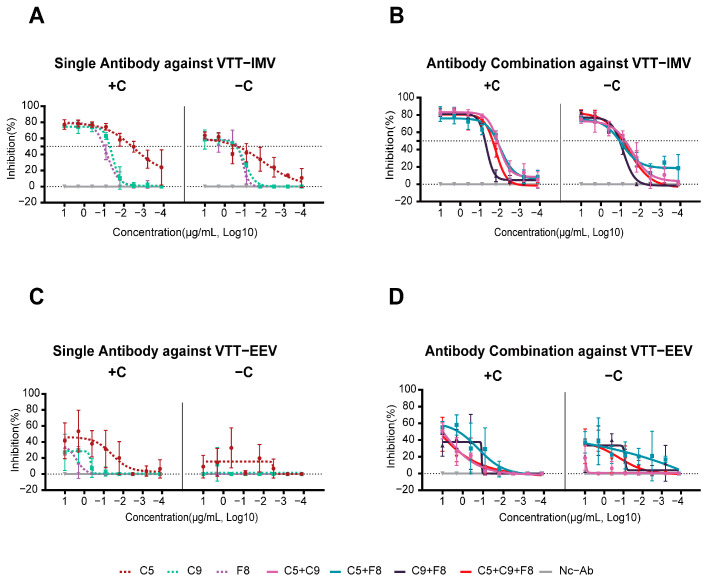
Neutralization activity of monoclonal antibodies and cocktails against the VTT with (+C) or without (−C) complement. (**A**) Neutralization of the VTT IMV by individual antibodies C5, C9, and F8. (**B**) Neutralization of the VTT IMV by the antibody combinations C5+C9, C5+F8, C9+F8, and C5+C9+F8. (**C**) Neutralization of the VTT EEV by individual antibodies. (**D**) Neutralization of the VTT EEV by antibody combinations.

The neutralization efficacy of mAbs against the VACV Tiantan IMV and EEV was systematically evaluated with the absence (−C) and presence (+C) of complement (Figure 3A,B). Individual antibodies and their combinations revealed distinct neutralization profiles.

In the absence of complement (Figure 3A, Table S2), the IC_50_ of the mAb C5 against the IMV was 233.8 ng/mL, with a maximum inhibition rate of 64%. Upon adding the complement, its IC_50_ decreased by 59.9-fold to 3.9 ng/mL, while the maximum inhibition rate increased to 77%. Similarly, for C9, the IC_50_ in the absence of complement was 268.6 ng/mL, with a maximum inhibition rate of 58%. The presence of complement reduced its IC_50_ by 5.3-fold to 51.1 ng/mL, and the maximum inhibition rate improved to 75%. F8 exhibited limited complement enhancement, the IC_50_ decreased by 2.2-fold from 227.3 ng/mL to 101.1 ng/mL with complement, and its maximum inhibition rate increased by about 22% from 63% to 77%.

For antibody combinations (Figure 3B), both the complement enhancement effect (CDE) and increasing inhibition rates against the IMV were observed. Notably, the combo achieved the highest maximum inhibition rate (86%) with a relatively low IC_50_ (23.3 ng/mL), suggesting that the antibodies may target distinct epitopes. Even in the absence of complement, the combo retained an IC_50_ of 79.3 ng/mL and a high maximum inhibition rate (81%).

No neutralizing activity against the EEV was detected in any of the three mAbs, even in the presence of complement (Figure 3C), confirming that the E8 mAbs specifically target the IMV.

### 3.7. Integrated Competitive Assays and Structural Analysis of Antibody–MPXV E8 or Antibody–VACV D8 Interactions

In order to clarify the spatial relationships and potential epitope overlaps among the C5, C9, and F8 mAbs, we conducted competitive binding assays and computational structural analysis of the E8 protein as a complex with each antibody.

**Figure 4:**
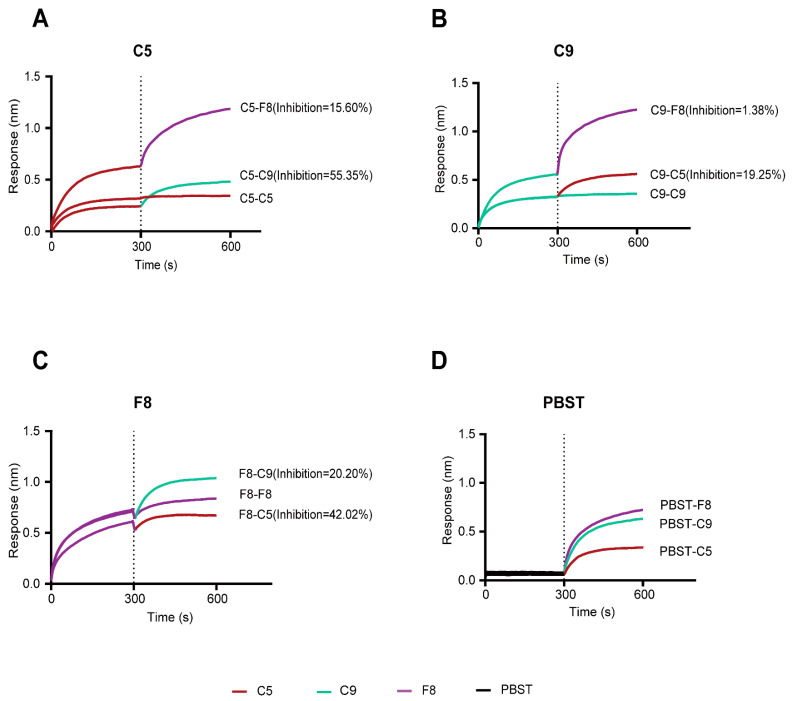
BLI-based competitive binding analysis of C5, C9, and F8 targeting the E8 antigen. (**A**) Competitive binding results with C5 as the primary antibody. (**B**) Competitive binding results with C9 as the primary antibody. (**C**) Competitive binding results with F8 as the primary antibody. (**D**) PBST as the buffer control. The inhibition percentage was defined as the percentage of secondary antibody binding suppressed by the first antibody.

The BLI measurements showed that when C5 was used as the primary antibody, it inhibited C9 and F8 by 55.35% and 15.60%, respectively (Figure 4A). In contrast, when C9 served as the primary antibody, it inhibited C5 by 19.25% and F8 by only 1.38% (Figure 4B). When F8 was the primary antibody, it inhibited C5 and C9 by 42.02% and 20.20%, respectively (Figure 4C). The data indicate that C5 and C9 may target highly overlapping or adjacent epitopes, while the C5 and F8 epitopes may partially overlap or not interfere with each other.

**Figure 5:**
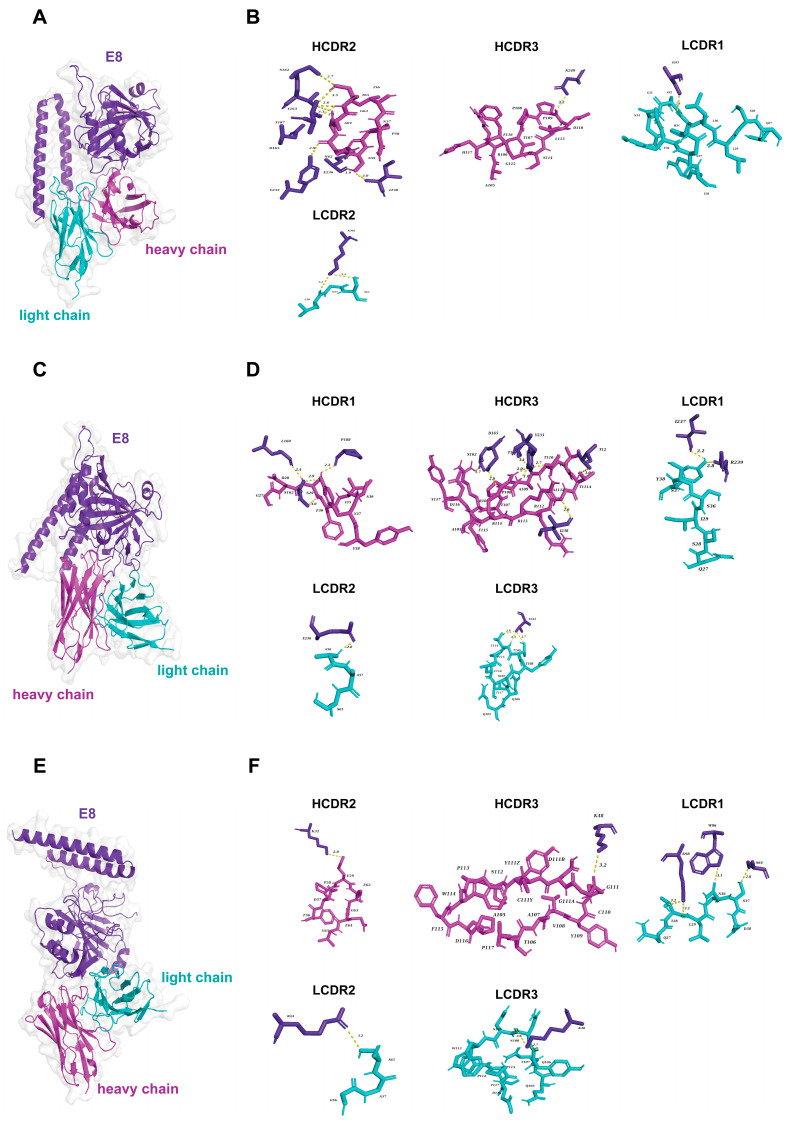
Model of the binding of C5, C9, and F8 to the MPXV E8 protein. (**A**) The overall structure of the C5–E8 complex, with the antibody heavy chain shown in magenta, the light chain in cyan, and the E8 antigen in purple-blue. (**B**) Detailed view of C5 binding interactions, highlighting the heavy chain complementarity-determining regions (HCDR2 and HCDR3) and the light chain complementarity-determining regions (LCDR1 and LCDR2). (**C**) The overall structure of the C9–E8 complex, with the antibody heavy chain shown in magenta, the light chain in cyan, and the E8 antigen in purple-blue. (**D**) Detailed view of C9 binding interactions, focusing on the HCDR1, HCDR3, and LCDR1–LCDR3 regions. (**E**) The overall structure of the F8–E8 complex, with the antibody heavy chain shown in magenta, the light chain in cyan, and the E8 antigen in purple-blue. (**F**) Detailed view of F8 binding interactions, highlighting the HCDR2, HCDR3, and LCDR1–LCDR3 regions.

**Figure 6:**
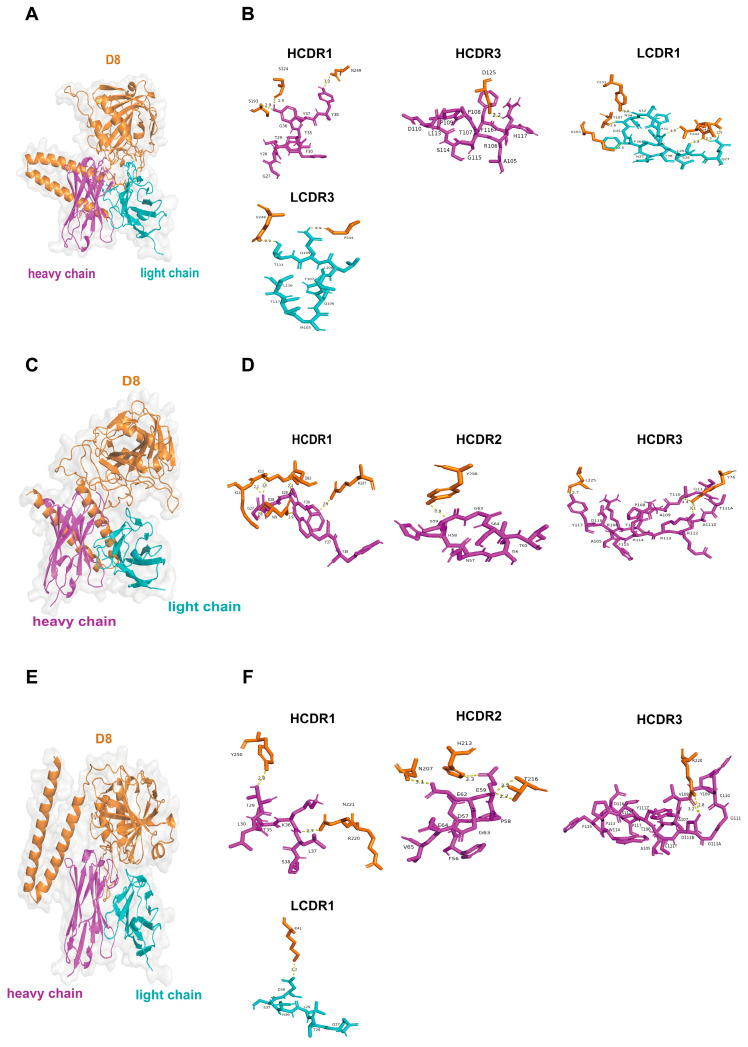
Model of the binding of C5, C9, and F8 to the VACV D8 protein. (**A**) The overall structure of the C5–D8 complex, with the antibody heavy chain shown in magenta, the light chain in cyan, and the D8 antigen in orange. (**B**) Detailed view of C5 binding interactions, highlighting the heavy chain complementarity-determining regions (HCDR1 and HCDR3) and the light chain complementarity-determining regions (LCDR1 and LCDR3). (**C**) The overall structure of the C9–D8 complex. (**D**) Detailed view of C9 binding interactions, focusing on the HCDR1, HCDR2, and HCDR3 regions. (**E**) The overall structure of the F8–D8 complex. (**F**) Detailed view of F8 binding interactions, highlighting the HCDR1, HCDR2, HCDR3, and LCDR1 regions.

Based on the crystal structure of the VACV D8 protein (strain Acam2000, PDB ID: 4E9O) resolved by Matho et al. [42], we employed AlphaFold 3 to predict three-dimensional structural models for the MPXV E8 and VTT D8 proteins. The structural alignment of the predicted models to the experimentally determined VACV D8 protein revealed root mean square deviation (RMSD) values of 0.208 Å for MPXV E8 and 0.180 Å for VTT D8, thereby validating the reliability and accuracy of our prediction models. The MPXV E8 and VACV D8 proteins comprise three functional domains: (1) residues 1–275 span the virion surface structure and mediate critical processes, including host receptor recognition and membrane fusion; (2) residues 276–294 constitute an α-helical domain that serves as a structural stabilizer through hydrophobic core packing; and (3) residues 295–304 function as intraviral core components essential for nucleocapsid assembly, genomic translocation, and viral replication initiation. For the C5 and F8 mAbs, their binding sites on the E8 protein are similar to those of D8. As shown in Figure 5 and Figure 6, the results of molecular docking indicate that C5 may predominantly bind to the virion surface region (SR) and intravirion region (IR) of E8 and D8, with 13 key interacting residues (Figure 5A,B and Figure 6A,B). Similarly, F8 exclusively binds to the SR with 12 key interacting residues (Figure 5E,F and Figure 6E,F). In contrast, mAb C9 exhibits distinct binding profiles to E8 and D8. C9 exclusively targets the SR of E8, engaging 18 critical residues (Figure 5C,D), while bridging both the SR and IR of D8 via 11 residues (Figure 6C,D). The difference in binding characteristics can partially explain the differences in the neutralization abilities of C9 against the MPXV and VACV. Detailed epitope information for the mAbs targeting the E8 and D8 proteins is listed in Table S3.

### 3.8. The Combo Shows Enhanced Protective Efficicency in Therapeutic Mouse Models

**Figure 7:**
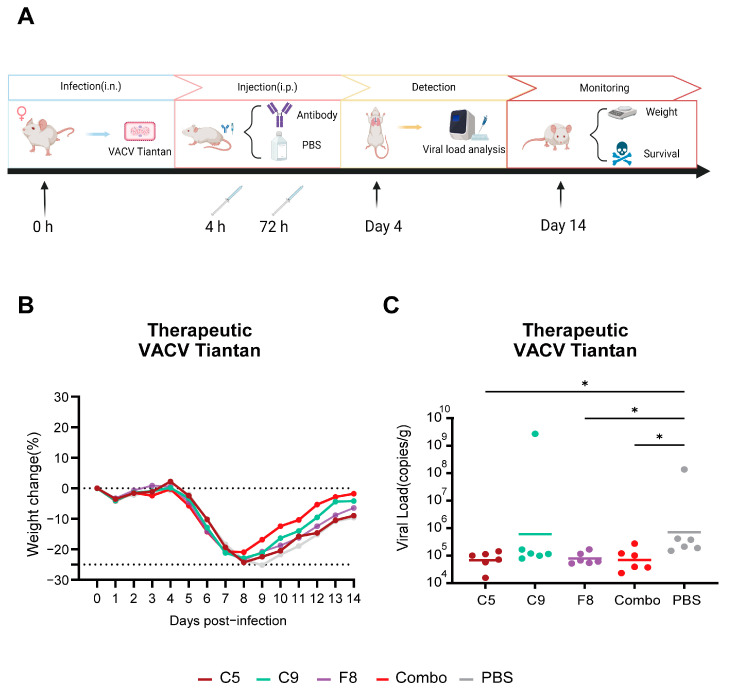
Therapeutic efficacy of mAbs against the VACV Tiantan in a mouse model. (**A**) Experimental design: treatment was administered at 4 and 72 h post-infection, with lung tissue collection on day 4. (**B**) Weight changes (%) in mice treated with individual antibodies (C5, C9, and F8) and the combo. (**C**) Viral loads (copies/g, geometric mean) in lung tissue on day 4 post-infection. Error bars represent standard deviations. Statistical analysis was performed using the Kruskal–Wallis test. * *p* < 0.05 versus the PBS group.

In order to evaluate the protective effects of E8-specific mAbs against VACV challenge, we carried out a therapeutic experiment in a mouse model. Groups of BALB/c mice received two doses of mAbs at 4 and 72 h post-infection (Figure 7A). Around day 8–9 after infection, when disease manifestation occurred, all groups reached their lowest body weight. The combo group exhibited the lowest body weight loss of 20.9% on day 8, which was lower than the individual antibody groups (C5: 24.3%; C9: 22.85%; F8: 23.56%) and the control group (25.19%), although there was no significant difference between the groups. However, the onset of recovery varied among the antibody-treated and control groups. The C5, C9, F8, and combo groups initiated weight recovery on day 9, whereas the control group commenced recovery on day 10 (Figure 7B). Although antibody treatments consistently conferred advantages in terms of reduced weight loss, the combo showed a more favorable outcome compared to individual antibody treatments. All groups demonstrated 100% survival rates, with no mortality events observed during the study.

Viral load quantification revealed significantly lower viral replication in the C5 group (6.7 × 10^4^ copies/g, *p* < 0.05), F8 group (7.1 × 10^4^ copies/g, *p* < 0.05), and combo group (6.5 × 10^4^ copies/g, *p* < 0.05) compared to the control group (5.0 × 10⁵ copies/g). In contrast, the C9 group (2.4 × 10⁵ copies/g, *p* ≥ 0.05) showed no statistically significant difference in viral replication compared to the control (Figure 7C). These results demonstrate that C5, F8, and the combo effectively suppress viral replication, whereas C9 monotherapy exhibits limited efficacy.

## 4. Discussion

Mpox has been declared a PHEIC twice since 2022, yet there are no effective treatments available. In this study, we identified three MPXV E8-specific mAbs—C5, C9, and F8—from volunteers receiving a recombinant VACV vaccine using an MPXV E8 probe. Notably, both the combo and C9 alone demonstrated potent cross-neutralization of the authentic MPXV in vitro, while all three antibodies effectively suppressed VACV replication. In a VACV-challenged murine model, these antibodies mitigated disease severity, as evidenced by a reduction in viral loads and weight loss, underscoring their therapeutic potential against the VACV and MPXV.

Previous studies have demonstrated that the smallpox vaccine can induce neutralizing antibodies against MPXV due to the cross-reactivity between the VACV and MPXV [20,43,44,45]. The VACV D8 protein, a key membrane protein, serves as a validated immunogen that elicits potent neutralizing antibodies. As the structural and functional homolog of VACV D8, MPXV E8 serves the same function through conserved domains. Gilchuk et al. demonstrated that D8-induced mAbs can neutralize the VACV and CPXV; however, their efficacy against the MPXV is considerably lower [18]. This difference is likely due to structural variations in their binding epitopes, with D8 being more conserved and accessible across orthopoxviruses while E8 may undergo conformational changes or epitope masking when interacting with host molecules, reducing its antibody recognition in the MPXV [42,46]. In contrast, our study revealed that the mAb C9 exhibits an ultrahigh binding affinity for both E8 and D8 proteins, achieving cross-neutralization of both the MPXV and VACV. Based on the predicted epitopes from structural analysis, C9 exclusively targets the SR of MPXV E8 (18 residues) while bridging both the SR and IR of D8. The difference in binding characteristics and high affinity can partially explain C9’s neutralization abilities against the MPXV. Strikingly, despite sharing comparable affinities to E8, neither C5 nor F8 exhibited cross-neutralization. They target the SR (nine residues) and IR (four residues) and the SR (twelve residues), respectively. Intriguingly, C9’s broader neutralization spectrum correlates with its engagement of both a higher number of critical residues and functionally conserved regions across orthopoxviruses. Antibodies targeting MPXV M1 effectively neutralize the replication-deficient MPXV in its IMV form [27]. In our findings, we further show that E8-specific antibodies exhibit potent neutralization against live MPXV. These findings collectively validate that MV-targeting antibodies can effectively inhibit the MPXV.

Studies have demonstrated that when two antibodies target overlapping epitopes, the high-affinity antibody can dominate the binding competition through either faster epitope occupation or slower dissociation kinetics. This principle is exemplified by Maroli’s discovery of the monoclonal antibody 8G3, which competitively inhibits ACE2 binding to the SARS-CoV-2 receptor-binding domain. Their work revealed that 8G3’s high-affinity advantage dictates its dominance in this molecular competition [47]. In this study, we found that C5 and C9 demonstrated asymmetric cross-inhibition due to their partially overlapping epitopes and ultrahigh affinities. C5 exhibited dominant inhibitory effects on C9, likely attributable to its superior binding avidity, and targets an epitope adjacent to C9’s binding site on E8. This spatial proximity allows C5 to create steric hindrance that blocks C9’s binding. Conversely, C9 showed only marginal inhibition of C5, suggesting that C9’s epitope is located at the peripheral region of C5’s binding site, which prevents effective competition.

Antigen–antibody binding stabilizes the free-state conformational repertoire of the antigen into distinct structural ensembles through a conformational selection mechanism, thereby preventing other antibodies from recognizing the original epitopes [48]. Similarly, antibody engagement can shift the antigen’s conformational equilibrium—as exemplified by IL-13 being locked into a receptor-inaccessible state upon VHH204 antibody binding—potentially obstructing the subsequent recognition of conformation-dependent epitopes by other antibodies through analogous mechanisms [49]. Despite F8’s moderate affinity, it achieved intermediate inhibition of C5. This inhibition may involve F8-induced conformational changes that destabilize C5’s binding interface. The low inhibition rate of C5 against F8 suggests that their respective epitopes may be spatially distant. Furthermore, minimal reciprocal inhibition between C9 and F8 supports the theory of spatially distinct epitopes.

We observed that complement-dependent enhancement potentiated mAb neutralization against the VACV, likely through enhancing effector functions, including antibody-dependent cellular cytotoxicity (ADCC) and complement-dependent cytotoxicity (CDC) [50]. However, the MPXV tested in our study demonstrated resistance to the complement-mediated neutralization mechanism. As we know, MPXV clade I encodes the mpox inhibitor of complement enzymes (MOPICE), a homolog of the VACV complement control protein (VCP). MOPICE can block the complement system by binding to C3b and C4b, acting as a cofactor for factor I to cleave these components into inactive fragments [51,52]. In contrast, clade II lacks the MOPICE entirely [53]. We observed that the addition of complement did not enhance the neutralizing activity of the antibodies against MPXV in vitro.

Our findings revealed a discrepancy between in vitro neutralization potency and the in vivo protective efficacy of mAbs. While the mAbs C5, C9, and F8 demonstrated robust VACV neutralization in vitro, their protection efficacy in vivo was limited. This functional divergence was also reported by Gilchuk et al. for D8-specific antibodies [18]. Their work showed that D8 antibodies alone—even as multi-epitope mixtures—failed to protect mice from mortality or weight loss significantly. However, combining D8 with other antibodies (e.g., anti-L1, A33, and B5) in the Mix6 cocktail provided complete protection against systemic infection. These findings suggest that D8-mediated systemic protection requires synergistic interactions with other antibodies. Although the three antibodies showed limited efficacy in reducing weight loss, C5 and F8 exhibited a higher ability to inhibit virus replication in vivo compared to C9. Matho et al. classified D8-targeting antibodies into four distinct groups based on epitope specificity: groups I and II are non-blocking antibodies that recognize epitopes distal to the chondroitin sulfate (CS) binding site, potentially mediating viral inhibition through complement-dependent mechanisms; group III comprises partially blocking antibodies with epitopes overlapping the crevice; and group IV antibodies directly occupy the core CS-binding cleft, completely abrogating the CS–E interaction while requiring complement for neutralization enhancement [42]. CS–E acts as VACV D8’s primary ligand, mediating viral attachment to host cells through high-affinity interactions between its dual-sulfated glycosaminoglycan structure and D8’s positively charged crevice. Based on the predicted epitopes, we found that C5 shares group I determinants (125D, 163K, and 187T), while C9 exhibits partial epitope overlapping with group I (9N, 13K, 14K, and 83D) and group IV (5L). F8 aligns with group IV residues (3Q, 41K, 220R, 221N, and 224K). As noted by Matho et al., group I antibodies targeting the N-terminal domain showed limited neutralization under complement-free conditions. This finding aligns with our observations. C5 and C9 exhibited limited neutralization capacities under complement-free conditions but displayed significantly enhanced neutralization activity with complement. This may also explain F8’s preserved neutralization capacity despite its lower binding affinity. Notably, while the antibody combination showed no significant synergistic effects in neutralization assays, the combo demonstrated additive benefits in reducing weight loss and suppressing viral loads.

A limitation of this study was the inherent uncertainties of computational structural predictions. The absence of resolved E8 crystal structures necessitates reliance on homology-based predictions, which may compromise prediction fidelity for MPXV E8 epitope mapping. Additionally, murine protection studies cannot fully recapitulate the true therapeutic effects of antibodies in humans, which may lead to variance in the outcomes of studies on antibody protection. Therefore, non-human primates should be used for further challenge studies. Finally, due to the current unavailability of MPXV clade I, neutralization assays were conducted using clade II. The neutralizing efficacy against clade I remains untested and further research will be conducted.

## 5. Conclusions

This study demonstrates the potential of three E8-specific mAbs in neutralizing both the MPXV and VACV, with the mAb C9 and combo showing especially strong anti-MPXV activity. Although exhibiting potent neutralizing activity in vitro, E8 antibodies provided partial protection in murine challenge models. This suggests that future research should prioritize the development of antibodies targeting more proteins of the MPXV. A rational combination strategy integrating these antibodies could achieve synergistic neutralization and robust protection and overcome potential viral escape.

## Figures and Tables

**Table 1:** Genetic characteristics of the heavy and light chains of mAbs.

		V Gene	D Gene	J Gene	CDR3	SHM
Heavy Chain	C5H	Homsap IGHV1-2*02 F	Homsap IGHD3-22*01 F	Homsap IGHJ4*02 F	ARTPPDLSGFH	4.86%
	C9H	Homsap IGHV4-34*01 F	Homsap IGHD6-6*01 F	Homsap IGHJ4*02 F	ARTPATITARRRFDY	5.26%
	F8H	Homsap IGHV1-24*01 F	Homsap IGHD2-21*02 F	Homsap IGHJ5*02 F	ATAVYCGGDCYSPWFDP	5.56%
Light Chain	C5L	Homsap IGKV2-28*01 F	-	Homsap IGKJ3*01 F	MQTLQTPLT	5.92%
	C9L	Homsap IGKV1-39*01 F	-	Homsap IGKJ4*01 F	QQSYTTPLT	2.94%
	F8L	Homsap IGKV3-15*01 F	-	Homsap IGKJ2*01 F	QQYNNWPPDT	2.94%

**Table 2:** IC_50_ values of antibodies and the combo against authentic MPXV.

Antibody	IC_50_ (μg/mL)	
	−C	+C
C5	>100	>100
C9	3.2	3.0
F8	>100	>100
Combo	0.4	2.8

+C: antibody with 0.1% guinea pig complement. −C: antibody without complement.

## Data Availability

Data will be made available upon request.

## Supplementary Materials

The following supporting information can be downloaded at: https://www.mdpi.com/article/10.3390/vaccines13050471/s1, Figure S1: E8-binding and VACV-neutralizing antibody activity in plasma from vaccinated individuals; Figure S2: The characterization of the E8 antibodies. Figure S3: Denaturing SDS-PAGE analysis of monoclonal antibodies; Table S1: Kinetic binding parameters of mAbs against MPXV E8 and VACV D8; Table S2: IC_50_ values and maximum inhibition rate of antibodies and combinations against VACV IMV; Table S3: Monoclonal antibodies C5, C9, and F8 target distinct potential epitopes on the E8 and D8 proteins.

## References

[1] Likos A.M., Sammons S.A., Olson V.A., Frace A.M., Li Y., Olsen-Rasmussen M., Davidson W., Galloway R., Khristova M.L., Reynolds M.G. (2005). A tale of two clades: Monkeypox viruses. J. Gen. Virol..

[2] Elsayed S., Bondy L., Hanage W.P. (2022). Monkeypox Virus Infections in Humans. Clin. Microbiol. Rev..

[3] Durski K.N., McCollum A.M., Nakazawa Y., Petersen B.W., Reynolds M.G., Briand S., Khalakdina A. (2018). Emergence of monkeypox in West Africa and Central Africa, 1970–2017. Wkly. Epidemiol. Rec..

[4] Alakunle E., Moens U., Nchinda G., Okeke M.I. (2020). Monkeypox Virus in Nigeria: Infection Biology, Epidemiology, and Evolution. Viruses.

[5] Adegboye O.A., Eugenia Castellanos M., Alele F.O., Pak A., Ezechukwu H.C., Hou K., Emeto T.I. (2022). Travel-Related Monkeypox Outbreaks in the Era of COVID-19 Pandemic: Are We Prepared?. Viruses.

[6] 2022–24 Mpox (Monkeypox) Outbreak: Global Trends [Internet]. https://worldhealthorg.shinyapps.io/mpx_global/#1_Overview.

[7] Masirika L.M., Udahemuka J.C., Schuele L., Ndishimye P., Otani S., Mbiribindi J.B., Marekani J.M., Mambo L.M., Bubala N.M., Boter M. (2024). Ongoing mpox outbreak in Kamituga, South Kivu province, associated with monkeypox virus of a novel Clade I sub-lineage, Democratic Republic of the Congo, 2024. Euro Surveill..

[8] Rivers C., Watson C., Phelan A.L. (2024). The Resurgence of Mpox in Africa. JAMA.

[9] Elsheikh R., Makram A.M., Vasanthakumaran T., Tomar S., Shamim K., Tranh N.D., Elsheikh S.S., Van N.T., Huy N.T. (2023). Monkeypox: A comprehensive review of a multifaceted virus. Infect. Med..

[10] Fink D.L., Callaby H., Luintel A., Beynon W., Bond H., Lim E.Y., Gkrania-Klotsas E., Heskin J., Bracchi M., Rathish B. (2023). Clinical features and management of individuals admitted to hospital with monkeypox and associated complications across the UK: A retrospective cohort study. Lancet Infect. Dis..

[11] Miller M.J., Cash-Goldwasser S., Marx G.E., Schrodt C.A., Kimball A., Padgett K., Noe R.S., McCormick D.W., Wong J.M., Labuda S.M. (2022). Severe Monkeypox in Hospitalized Patients—United States, August 10–October 10, 2022. MMWR Morb. Mortal. Wkly. Rep..

[12] Mitjà O., Alemany A., Marks M., Lezama Mora J.I., Rodríguez-Aldama J.C., Torres Silva M.S., Corral Herrera E.A., Crabtree-Ramirez B., Blanco J.L., Girometti N. (2023). Mpox in people with advanced HIV infection: A global case series. Lancet.

[13] Higgins E., Ranganath N., Mehkri O., Majeed A., Walker J., Spivack S., Bhaimia E., Benamu E., Hand J., Keswani S. (2023). Clinical features, treatment, and outcomes of mpox in solid organ transplant recipients: A multicenter case series and literature review. Am. J. Transplant..

[14] Siegrist E.A., Sassine J. (2023). Antivirals With Activity Against Mpox: A Clinically Oriented Review. Clin. Infect. Dis..

[15] National Institutes of Health (NIH) [Internet] (2024). The Antiviral Tecovirimat Is Safe but Did Not Improve Clade I Mpox Resolution in Democratic Republic of the Congo. https://www.nih.gov/news-events/news-releases/antiviral-tecovirimat-safe-did-not-improve-clade-i-mpox-resolution-democratic-republic-congo.

[16] Smith T.G., Gigante C.M., Wynn N.T., Matheny A., Davidson W., Yang Y., Condori R.E., O’Connell K., Kovar L., Williams T.L. (2023). Tecovirimat Resistance in Mpox Patients, United States, 2022–2023. Emerg. Infect. Dis..

[17] Pantaleo G., Correia B., Fenwick C., Joo V.S., Perez L. (2022). Antibodies to combat viral infections: Development strategies and progress. Nat. Rev. Drug Discov..

[18] Gilchuk I., Gilchuk P., Sapparapu G., Lampley R., Singh V., Kose N., Blum D.L., Hughes L.J., Satheshkumar P.S., Townsend M.B. (2016). Cross-Neutralizing and Protective Human Antibody Specificities to Poxvirus Infections. Cell.

[19] Alakunle E., Kolawole D., Diaz-Cánova D., Alele F., Adegboye O., Moens U., Okeke M.I. (2024). A comprehensive review of monkeypox virus and mpox characteristics. Front. Cell. Infect. Microbiol..

[20] Li E., Guo X., Hong D., Gong Q., Xie W., Li T., Wang J., Chuai X., Chiu S. (2023). Duration of humoral immunity from smallpox vaccination and its cross-reaction with Mpox virus. Signal Transduct. Target. Ther..

[21] Rao A.K. (2022). Use of JYNNEOS (Smallpox and Monkeypox Vaccine, Live, Nonreplicating) for Preexposure Vaccination of Persons at Risk for Occupational Exposure to Orthopoxviruses: Recommendations of the Advisory Committee on Immunization Practices—United States, 2022. MMWR Morb. Mortal. Wkly. Rep..

[22] Ramírez J.C., Tapia E., Esteban M. (2002). Administration to mice of a monoclonal antibody that neutralizes the intracellular mature virus form of vaccinia virus limits virus replication efficiently under prophylactic and therapeutic conditions. J. Gen. Virol..

[23] Wolffe E.J., Vijaya S., Moss B. (1995). A myristylated membrane protein encoded by the vaccinia virus L1R open reading frame is the target of potent neutralizing monoclonal antibodies. Virology.

[24] Hsiao J.C., Chung C.S., Chang W. (1999). Vaccinia virus envelope D8L protein binds to cell surface chondroitin sulfate and mediates the adsorption of intracellular mature virions to cells. J. Virol..

[25] Law M., Smith G.L. (2001). Antibody neutralization of the extracellular enveloped form of vaccinia virus. Virology.

[26] Lin C.L., Chung C.S., Heine H.G., Chang W. (2000). Vaccinia virus envelope H3L protein binds to cell surface heparan sulfate and is important for intracellular mature virion morphogenesis and virus infection in vitro and in vivo. J. Virol..

[27] Qu Y., Tai W., Ma E., Jiang Q., Fan M., Xiao W., Tian C., Liu Y., Liu J., Wang X. (2025). Generation and characterization of neutralizing antibodies against M1R and B6R proteins of monkeypox virus. Nat. Commun..

[28] Zhao R., Wu L., Sun J., Liu D., Han P., Gao Y., Zhang Y., Xu Y., Qu X., Wang H. (2024). Two noncompeting human neutralizing antibodies targeting MPXV B6 show protective effects against orthopoxvirus infections. Nat. Commun..

[29] Li M., Chen J., Wang F., Kuang J., Peng Y., Asghar S., Zhao W., Yang Y., Shen C. (2025). Bispecific antibodies targeting MPXV A29 and B6 demonstrate efficacy against MPXV infection. J. Virol..

[30] Li M., Ren Z., Wang Y., Jiang Y., Yang M., Li D., Chen J., Liang Z., Lin Y., Zeng Z. (2023). Three neutralizing mAbs induced by MPXV A29L protein recognizing different epitopes act synergistically against orthopoxvirus. Emerg. Microbes Infect..

[31] Zhou B., Wang H., Cheng L., Zhao C., Zhou X., Liao X., Ge X., Liu L., Lu X., Ju B. (2023). Two long-lasting human monoclonal antibodies cross-react with monkeypox virus A35 antigen. Cell Discov..

[32] Zhu W., Zhang M., Zhang M., Jing R., Zhou J., Cao H., Liu C., Zhu H., Ghonaim A.H., Rouby S.R. (2024). The Generation and Characterization of Monoclonal Antibodies against the MPXV A29L Protein. Viruses.

[33] Sagdat K., Batyrkhan A., Kanayeva D. (2024). Exploring monkeypox virus proteins and rapid detection techniques. Front. Cell. Infect. Microbiol..

[34] Tiller T., Meffre E., Yurasov S., Tsuiji M., Nussenzweig M.C., Wardemann H. (2008). Efficient generation of monoclonal antibodies from single human B cells by single cell RT-PCR and expression vector cloning. J. Immunol. Methods.

[35] Kong L., Ju B., Chen Y., He L., Ren L., Liu J., Hong K., Su B., Wang Z., Ozorowski G. (2016). Key gp120 Glycans Pose Roadblocks to the Rapid Development of VRC01-Class Antibodies in an HIV-1-Infected Chinese Donor. Immunity.

[36] Accurate Structure Prediction of Biomolecular Interactions with AlphaFold 3 | Nature [Internet]. https://www.nature.com/articles/s41586-024-07487-w.

[37] Delano W.L. (2002). PyMOL: An Open-Source Molecular Graphics Tool. http://www.researchgate.net/publication/313391141_PyMOL_An_Open-Source_Molecular_Graphics_Tool.

[38] Lavinder J.J., Wine Y., Giesecke C., Ippolito G.C., Horton A.P., Lungu O.I., Hoi K.H., DeKosky B.J., Murrin E.M., Wirth M.M. (2014). Identification and characterization of the constituent human serum antibodies elicited by vaccination. Proc. Natl. Acad. Sci. USA.

[39] Victora G.D., Nussenzweig M.C. (2012). Germinal centers. Annu. Rev. Immunol..

[40] Klein F., Diskin R., Scheid J.F., Gaebler C., Mouquet H., Georgiev I.S., Pancera M., Zhou T., Incesu R.-B., Fu B.Z. (2013). Somatic mutations of the immunoglobulin framework are generally required for broad and potent HIV-1 neutralization. Cell.

[41] Robbiani D.F., Gaebler C., Muecksch F., Lorenzi J.C.C., Wang Z., Cho A., Agudelo M., Barnes C.O., Gazumyan A., Finkin S. (2020). Convergent antibody responses to SARS-CoV-2 in convalescent individuals. Nature.

[42] Matho M.H., de Val N., Miller G.M., Brown J., Schlossman A., Meng X., Crotty S., Peters B., Xiang Y., Hsieh-Wilson L.C. (2014). Murine anti-vaccinia virus D8 antibodies target different epitopes and differ in their ability to block D8 binding to CS-E. PLoS Pathog..

[43] Li M., Guo Y., Deng Y., Gao W., Huang B., Yao W., Zhao Y., Zhang Q., Huang M., Liu M. (2024). Long-lasting humoral and cellular memory immunity to vaccinia virus Tiantan provides pre-existing immunity against mpox virus in Chinese population. Cell Rep..

[44] Huang Q., Wang Y., Zhao T., Wang Y., Wang X., Li S., Su W., Ren X., Zhang X., Liu J. (2023). Examination of the cross-reactivity between vaccinia virus Tiantan strain and monkeypox virus. J. Virol. Methods.

[45] Marchi S., Piccini G., Cantaloni P., Guerrini N., Zannella R., Coluccio R., Benincasa L., Solfanelli N., Remarque E.J., Viviani S. (2024). Evaluation of monkeypox- and vaccinia-virus neutralizing antibodies before and after smallpox vaccination: A sero-epidemiological study. J. Med. Virol..

[46] Das R., Bhattarai A., Karn R., Tamang B. (2024). Computational investigations of potential inhibitors of monkeypox virus envelope protein E8 through molecular docking and molecular dynamics simulations. Sci. Rep..

[47] Maroli N. (2023). Riding the Wave: Unveiling the Conformational Waves from RBD of SARS-CoV-2 Spike Protein to ACE2. J. Phys. Chem. B.

[48] da Silva P.C.C., Martinez L. (2024). Extended Conformational Selection in the Antigen-Antibody Interaction of the PfAMA1 Protein. J. Phys. Chem. B.

[49] Walker K., Baravalle R., Holyfield R., Kalms J., Wright H., Seewooruthun C., Muskett F.W., Scott-Tucker A., Merritt A., Henry A. (2023). Identification and characterisation of anti-IL-13 inhibitory single domain antibodies provides new insights into receptor selectivity and attractive opportunities for drug discovery. Front. Immunol..

[50] Carroll M.C. (2004). The complement system in regulation of adaptive immunity. Nat. Immunol..

[51] Hudson P.N., Self J., Weiss S., Braden Z., Xiao Y., Girgis N.M., Emerson G., Hughes C., Sammons S.A., Isaacs S.N. (2012). Elucidating the role of the complement control protein in monkeypox pathogenicity. PLoS ONE.

[52] Liszewski M.K., Leung M.K., Hauhart R., Buller R.M.L., Bertram P., Wang X., Rosengard A.M., Kotwal G.J., Atkinson J.P. (2006). Structure and regulatory profile of the monkeypox inhibitor of complement: Comparison to homologs in vaccinia and variola and evidence for dimer formation. J. Immunol..

[53] Li Y., Hou J., Sun Z., Hu J., Thilakavathy K., Wang Y., Shao Z., Lu Y., Wang W., Xiong C. (2023). Monkeypox virus 2022, gene heterogeneity and protein polymorphism. Signal Transduct. Target. Ther..

