# Supplementary material for: Isolation and Characterization of E8 Monoclonal Antibodies from Donors Vaccinated with Recombinant Vaccinia Vaccine with Efficient Neutralization of Authentic Monkeypox Virus
Source: Vaccines (Basel). 2025 Apr 27;13(5):471. doi: 10.3390/vaccines13050471 (PMC12116032; doi:10.3390/vaccines13050471)
Supplement: Supplementary file 1 [file vaccines-13-00471-s001.zip › vaccines-3596848-supplementary.pdf]

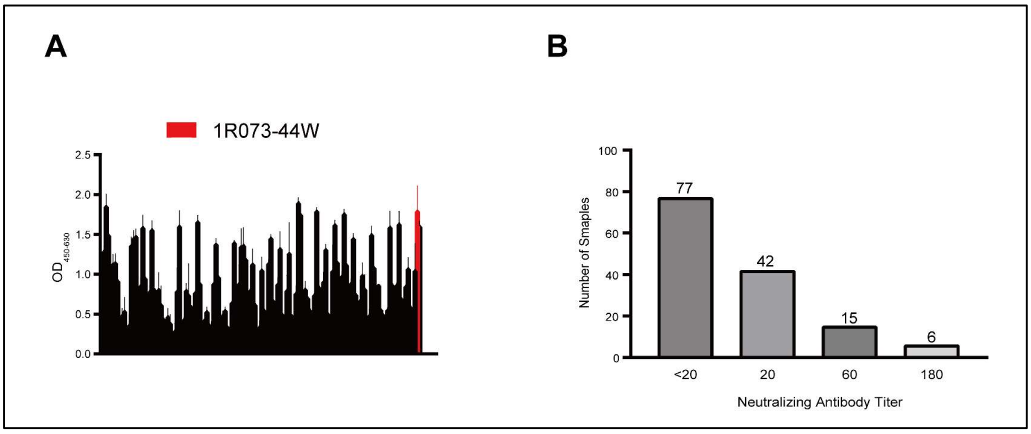

Figure S1. E8-binding and VACV-neutralizing antibody activity in plasma from vaccinated individuals. (**A**) E8-binding antibody activity in plasma from vaccinated individuals. (**B**) Neutralizing antibody titer for plasma from vaccinated individuals.

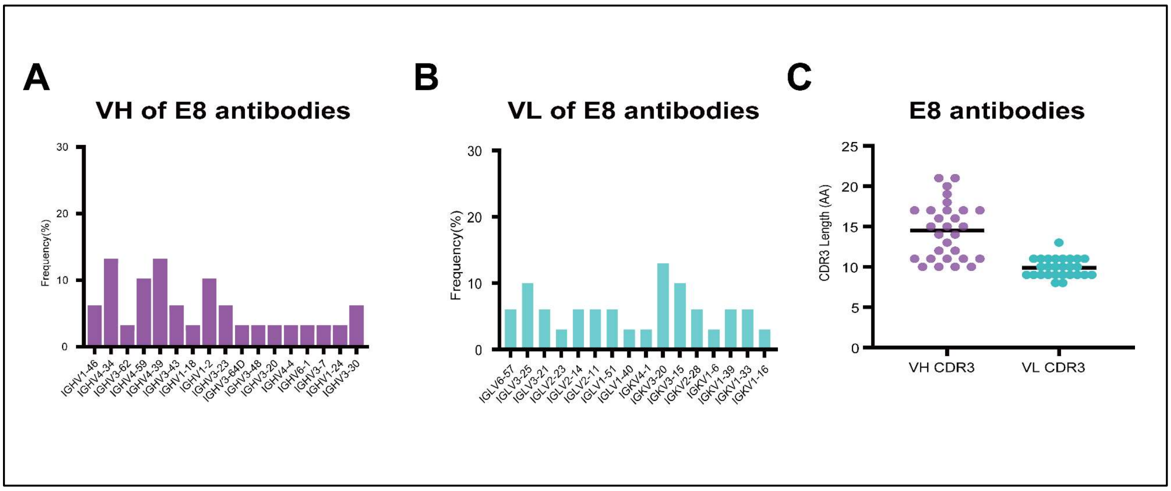

Figure S2. The characterization of the E8 antibodies. (**A**) Distribution of human IGVH gene frequencies in E8 antibodies. (**B**) Distribution of human IGVL gene frequencies in E8 antibodies. (**C**) CDR3 length at IGVH and IGVL in E8 antibodies.

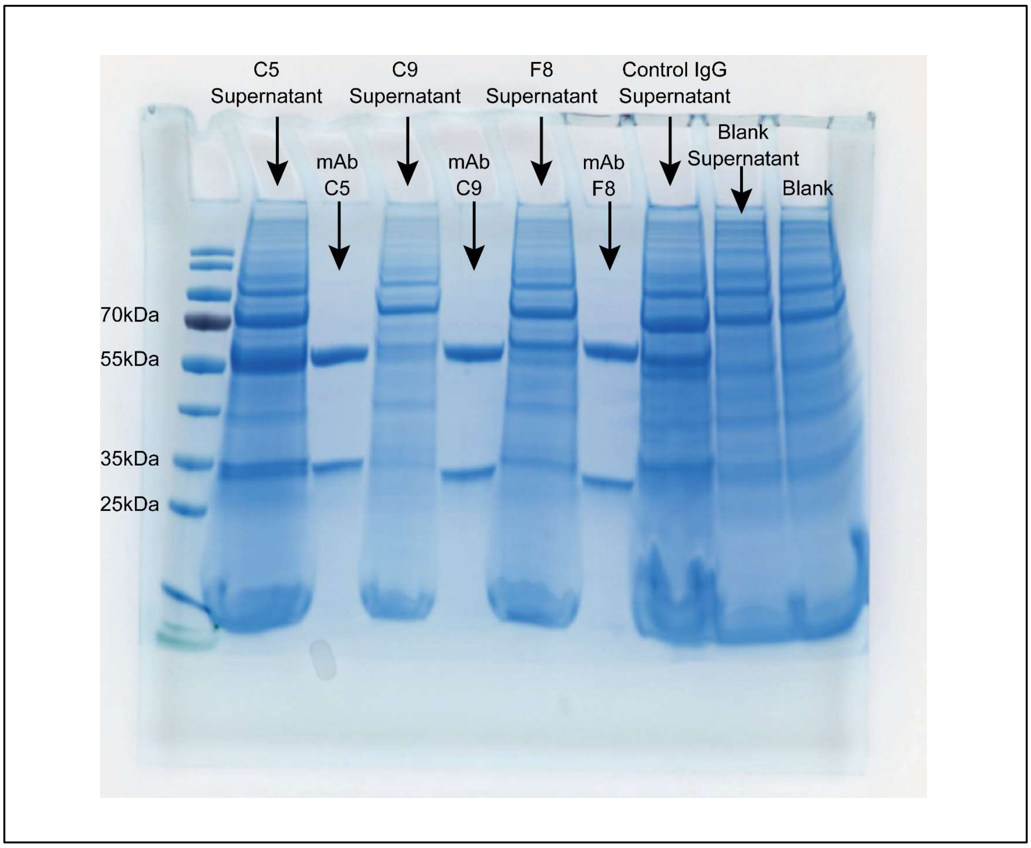

Figure S3. Denaturing SDS-PAGE Analysis of Monoclonal Antibodies. The lanes show proteins from the supernatants of C5, C9, F8, and control IgG samples and the corresponding monoclonal antibodies (mAb C5, mAb C9, and mAb F8). Molecular weight markers (kDa) are shown on the left, with arrows pointing to the specific bands for each sample.

Table S1. Kinetic Binding Parameters of mAbs against MPXV E8 and VACV D8.

	MPXV E8			VACV D8		
**Antibody**	**KD (M)**	**Kon (1/Ms)**	**Koff (1/s)**	**KD (M)**	**Kon (1/Ms)**	**Koff (1/s)**
C5	$<1\times10^{-12}$	1.03×10^{6}	$<1\times10^{-7}$	$<1\times10^{-12}$	8.90×10^{5}	$<1\times10^{-7}$
C9	$<1\times10^{-12}$	4.86×10^{5}	$<1\times10^{-7}$	$<1\times10^{-12}$	3.42×10^{5}	$<1\times10^{-7}$
F8	4.79×10^{-9}	8.36×10^{4}	5.96×10^{-4}	3.18×10^{-8}	6.24×10^{4}	1.99×10^{-3}

Table S2. IC_{50} Values and maximum inhibition rate of Antibodies and Combinations against VACV IMV.

**Antibody**	**IC_{50} (ng/mL)**		**Change(folds)**	**maximum inhibition rate(%)**	
	-C	+C		-C	+C
C5	233.8	3.9	59.9	64	77
C9	268.6	51.1	5.3	58	75
F8	227.3	101.1	2.2	63	77
C5+C9	59.5	13.5	4.4	75	82
C5+F8	115.8	14.3	8	76	81
C9+F8	114.8	56.4	2	82	81
Combo	79.3	23.3	3.4	81	86

+C: Antibody with 0.1% guinea pig complement.

-C: Antibody without complement.

Table S3. Monoclonal antibodies C5, C9, and F8 target distinct potential epitopes on the E8 and D8 proteins.

E8 epitopes	Antibody			D8 epitopes	Antibody		
	C5	C9	F8		C5	C9	F8
12T	-	+	-	3Q	-	-	+
17S	-	+	-	4Q	-	+	-
19T	-	+	-	5L	-	+	-
21L	-	+	-	9N	-	+	-
22K	-	+	-	13K	-	+	-
23T	-	+	-	14K	-	+	-
32K	-	-	+	41K	-	-	+
37Q	-	-	+	76Y	-	+	-
44R	-	-	+	83D	-	+	-
46N	-	-	+	124S	+	-	-
48K	-	-	+	125D	+	-	-
55G	-	+	-	163K	+	-	-
64S	-	-	+	187T	+	-	-
96W	-	-	+	193S	+	-	-
98K	-	-	+	207N	-	-	+
102S	-	-	+	208H	-	-	+
103S	-	-	+	213H	-	-	+
104Y	-	-	+	216T	-	-	+
105E	-	-	+	220R	-	-	+
125D	+	-	-	221N	-	-	+
126H	+	+	-	224K	-	-	+
127K	+	-	-	225L	-	+	-
145N	-	-	+	243T	+	-	-
147S	-	-	+	244P	+	-	-
160L	-	+	-	247R	-	+	-
162S	-	+	-	248E	+	-	-
163T	+	-	-	249N	+	-	-
165D	+	-	-	250Y	-	-	+
183T	+	-	-	254W	-	+	-
187T	-	+	-	261T	-	-	+
188P	-	+	-	265Y	-	-	+
233Y	-	+	-	296R	+	-	-
236E	+	-	-	298Y	-	+	-
237I	-	+	-				
238I	+	+	-				
239R	-	+	-				
243T	-	+	-				
249N	+	-	-				
295S	+	-	-				
299S	+	-	-				
302K	+	-	-				
303Q	+	-	-				

+: Predicted antibody-epitope binding.

-: No predicted binding.
